# Improving Prognostic Accuracy of MASCC Score with Lactate and CRP Measurements in Febrile Neutropenic Patients

**DOI:** 10.3390/diagnostics15151922

**Published:** 2025-07-31

**Authors:** Efe Kanter, Ecem Ermete Güler, Süleyman Kırık, Tutku Duman Şahan, Melisa Buse Baygın, Emine Altınöz, Ejder Saylav Bora, Zeynep Karakaya

**Affiliations:** 1 Department of Emergency Medicine, Faculty of Medicine, Izmir Katip Çelebi University, 35620 Izmir, Turkey; ecemermete@hotmail.com (E.E.G.); kiriksuleyman2107@outlook.com (S.K.); drmelisabuse@gmail.com (M.B.B.); eminealtinoz@gmail.com (E.A.); ejdersaylav.bora@ikc.edu.tr (E.S.B.); zeynepkarakaya76@hotmail.com (Z.K.); 2 Department of Emergency Medicine, Cesme Alper Cizgenakat State Hospital, 35930 Izmir, Turkey; tutkuduman75@gmail.com

**Keywords:** febrile neutropenia, MASCC score, risk stratification

## Abstract

**Objectives**: Febrile neutropenia is a common oncologic emergency with significant morbidity and mortality. Although the MASCC (Multinational Association for Supportive Care in Cancer) score is widely used for risk stratification, its limited sensitivity and lack of laboratory parameters reduce its prognostic utility. This study aimed to evaluate whether incorporating serum lactate and CRP measurements into the MASCC score enhances its predictive performance for hospital admission and the 30-day mortality. **Methods**: This retrospective diagnostic accuracy study included adult patients diagnosed with febrile neutropenia in the emergency department of a tertiary care hospital between January 2021 and December 2024. The original MASCC score was calculated, and three modified models were derived: the MASCC-L (lactate/MASCC), MASCC-C (CRP/MASCC) and MASCC-LC models (CRP × lactate/MASCC). The predictive accuracy for hospital admission and the 30-day all-cause mortality was assessed using ROC analysis. **Results**: A total of 269 patients (mean age: 67.6 ± 12.4 years) were included; the 30-day mortality was 3.0%. The MASCC-LC model demonstrated the highest discriminative ability for mortality prediction (area under the curve (AUC): 0.995; sensitivity: 100%; specificity: 98%). For hospital admission prediction, the MASCC-C model had the highest specificity (81%), while the MASCC-LC model showed the best balance of sensitivity and specificity (both 73%). All the modified models outperformed the original MASCC score regarding both endpoints. **Conclusions**: Integrating lactate and CRP measurements into the MASCC score significantly improves its prognostic accuracy for both mortality and hospital admission in febrile neutropenic patients. The MASCC-LC model, relying on only three objective parameters, may serve as a practical and efficient tool for early risk stratification in emergency settings.

## 1. Introduction

Febrile neutropenia (FN) is a common clinical condition in patients undergoing treatment for malignancy and is associated with significant morbidity and mortality. The incidence of FN varies depending on the cancer type and chemotherapy regimen, and the condition is associated with a mortality rate ranging from 5% to 20% in high-risk groups [1,2,3]. If not promptly recognized and managed, FN can progress rapidly to severe sepsis, septic shock, multi-organ failure and death, particularly in patients with hematologic malignancies or profound neutropenia [4,5]. Therefore, early identification and accurate risk stratification of high-risk patients is essential to guide triage decisions, initiate timely antibiotic therapy and optimize overall patient management in emergency care settings where rapid triage is essential [4,6].

One of the most widely used tools for this purpose is the MASCC (Multinational Association for Supportive Care in Cancer) risk index, developed to identify low-risk patients who may be suitable for outpatient treatment [7,8]. The MASCC score is based on clinical parameters such as patients’ symptom severity, vital signs, age and comorbidities, and patients with a score of 21 or above are considered to be at a low risk for adverse outcomes [9]. However, the score has certain limitations, including its reliance on subjective components, exclusion of laboratory data and insufficient sensitivity in emergency department settings [10,11,12].

Beyond the MASCC score, other scoring systems such as the Clinical Index of Stable Febrile Neutropenia (CISNE), qSOFA and Pitt bacteremia score have also been proposed to predict adverse outcomes in FN patients [13]. However, the MASCC score remains the most widely used due to its simplicity and broad validation across oncology populations. Nevertheless, it was originally designed to identify low-risk patients suitable for outpatient management, and its performance significantly declines in critically ill patients, those with hematologic malignancies or patients presenting with a systemic inflammatory response [10,11,14]. In particular, patients with borderline scores of between 15 and 21 are often clinically heterogeneous, and their actual risk may be underestimated due to the lack of consideration of objective inflammatory markers. This clinical gap has led researchers to explore whether integrating objective biomarkers reflecting the inflammatory burden or tissue hypoperfusion could enhance the prognostic accuracy.

In response to these limitations, various studies have proposed modifications to enhance the prognostic performance of the MASCC score. In particular, the integration of biomarkers into clinical scoring systems has led to the development of new approaches capable of predicting complications and mortality with greater accuracy [3,8,9]. Biomarkers such as lactate and C-reactive protein (CRP) reflect different but complementary physiological processes. Lactate is a surrogate for tissue hypoperfusion and early organ dysfunction, while CRP is a well-established indicator of systemic inflammation [15]. Their incorporation into risk models has been shown to improve prognostic discrimination, especially in acute care settings [16]. However, most of these modifications have not been standardized, and their applicability in emergency department practice remains limited [14,17].

The present study aims to evaluate the prognostic utility of MASCC-based models that incorporate lactate and CRP measurements and to compare their performance with the original MASCC score in predicting the 30-day mortality and hospital admission. We hypothesize that the integration of these biomarkers into the MASCC framework will yield improved prognostic accuracy, thereby enhancing risk stratification and informing clinical decisions in emergency settings.

## 2. Materials and Methods

### 2.1. Study Design and Setting

This study was designed as a retrospective and observational diagnostic accuracy study. The data were obtained from the emergency department records of a tertiary care training and research hospital. Ethical approval was granted by the local Health Research Ethics Committee (Decision No.: 0342; Date: 10 April 2025).

Given the retrospective and observational nature of this study, no formal sample size calculation was performed a priori; instead, all the eligible cases were included to enhance the statistical power and minimize selection bias.

### 2.2. Study Population

Patients aged 18 years or older who presented to the emergency department between January 2021 and December 2024 and were diagnosed with febrile neutropenia were included in this study. Febrile neutropenia was defined as a body temperature of ≥38.0 °C accompanied by an absolute neutrophil count of <500/mm^3^.

Patients were excluded if they were under 18 years of age, had missing clinical data necessary for MASCC score calculation, lacked lactate or CRP measurements at presentation, had unavailable 30-day mortality data or presented multiple times (only the first admission was included for patients with repeated visits).

### 2.3. Data Collection

The data were retrospectively obtained through the hospital information management system. Demographic characteristics (age and sex), vital signs at presentation, laboratory values (lactate and CRP), clinical parameters used for MASCC scoring and outcome data were recorded.

The serum lactate levels were measured using a blood gas analyzer (Radiometer ABL800 FLEX, Copenhagen, Denmark), and the CRP levels were measured using an immunoturbidimetric method on a Roche Cobas c702 analyzer (Roche Diagnostics, Basel, Switzerland). Both measurements were performed within the first 30 min after the patient’s arrival at the emergency department. Although both the CRP and lactate levels were measured within the first 30 min of emergency department arrival, we acknowledge that their values may have fluctuated depending on the disease progression and treatment initiation. Therefore, the use of these biomarkers as early prognostic indicators is the most valid when measured prior to antibiotic or fluid resuscitation, as was the case in our protocol.

#### MASCC and Modified MASCC Models

For each patient, the original MASCC score was calculated based on seven clinical criteria defined in the literature:

- No severe symptoms → 5 points (mild or moderate symptoms → 3 points).
- No hypotension (systolic blood pressure of ≥90 mmHg) → 5 points.
- No chronic obstructive pulmonary disease (COPD) → 4 points.
- A hematologic malignancy without a solid tumor or fungal infection → 4 points.
- No signs of dehydration → 3 points.
- An outpatient status at the onset of febrile neutropenia → 3 points.
- An age < 60 years → 2 points.

The total maximum score was 26, and patients with a score of 21 or higher were considered to be at a low risk. In this study, to modify the MASCC score, the serum lactate (mmol/L) and C-reactive protein (CRP; mg/L) levels measured at admission were integrated to create three different models:

- **MASCC-L model:** The ratio of the lactate level to the MASCC score (lactate/MASCC).
- **MASCC-C model:** The ratio of the CRP level to the MASCC score (CRP/MASCC).
- **MASCC-LC model:** The ratio of the product of the CRP and lactate levels to the MASCC score (CRP × lactate/MASCC).

Cases with missing outcome data or CRP or lactate measurements were excluded from the analysis. No imputation methods were applied for missing values.

### 2.4. Outcome Measures

#### 2.4.1. Primary Outcome

The primary outcome was the patient’s disposition after presentation, categorized as discharge, admission to a general ward or admission to the intensive care unit.

#### 2.4.2. Secondary Outcome

The secondary outcome was the all-cause mortality within 30 days following presentation. Disposition data were obtained from the hospital information system and mortality data were confirmed using both the hospital records and the national death reporting system.

### 2.5. Statistical Analysis

All the statistical analyses were conducted using IBM SPSS Statistics version 26.0 (IBM Corp., Armonk, NY, USA). Continuous variables were presented as the mean (standard deviation) or median (minimum–maximum), depending on the distribution characteristics. Categorical variables were expressed as numbers and percentages. For comparisons of the continuous variables between the two groups, the Mann–Whitney U test was used; for comparisons across more than two groups, the Kruskal–Wallis test was applied. The categorical variables were compared using the chi-square test.

The prognostic performance of the original MASCC score and its modified versions was assessed for two clinical outcomes: hospital admission (defined as ward or ICU admission versus discharge) and the 30-day all-cause mortality. Prior to ROC (Receiver Operating Characteristic) curve analysis, the original MASCC score was inversely transformed (1/MASCC) to ensure that higher scores reflected a higher clinical risk, consistent with the other models. The area under the curve (AUC) was calculated for each scoring model.

The optimal cut-off values were determined using Youden’s Index (sensitivity + specificity − 1), and the corresponding sensitivity, specificity, positive predictive value (PPV) and negative predictive value (NPV) were reported. To calculate the 95% confidence intervals and standard errors of the AUC values, non-parametric bootstrap resampling with 1000 iterations was applied. In each iteration, a random sample of the same size as the original dataset was drawn with replacement, and the AUC was recalculated to estimate the variability in the model performance.

A *p*-value of <0.05 was considered statistically significant.

## 3. Results

A total of 269 patients diagnosed with febrile neutropenia were included in this study. The mean age of the study population was 67.6 (12.4) years. The mean MASCC score was 20.4 (4.4), the mean CRP level was 138.5 (107.8) mg/L and the mean lactate level was 2.6 (2.5) mmol/L. At initial presentation, 75 patients (27.9%) were discharged, 150 patients (55.8%) were admitted to the general ward and 44 patients (16.4%) required admission to the intensive care unit (ICU). Mortality was observed in eight patients (3.0%) (Table 1).

Additionally, the gender distribution was 152 males (56.5%) and 117 females (43.5%). The most common underlying malignancy was acute myeloid leukemia (31.2%), followed by non-Hodgkin lymphoma (22.7%) and multiple myeloma (15.6%). A total of 236 patients (87.7%) had hematological malignancies, while the remainder had solid tumors receiving myelosuppressive chemotherapy.

Group-wise comparisons of the clinical variables are summarized in Table 2. The comparison of the clinical parameters across the disposition groups revealed a statistically significant difference in the MASCC scores, CRP levels and lactate levels (*p* < 0.001 for all). Patients admitted to the ICU had notably lower MASCC scores and higher levels of inflammatory markers compared to the other groups. However, there was no statistically significant difference in the mean age across the disposition groups (*p* = 0.737). Similarly, when mortality was considered as the outcome, significant differences were observed in the MASCC scores, CRP levels and lactate levels (*p* < 0.001), while age did not differ significantly between the survivors and non-survivors (*p* = 0.991).

Additionally, the patients who died had significantly higher lactate and CRP levels compared to the survivors. Among those who died, the mean MASCC score was 11.0 (2.6), the mean CRP level was 241.8 (38.4) mg/L and the mean lactate level was 11.5 (8.4) mmol/L.

**Figure 1:**
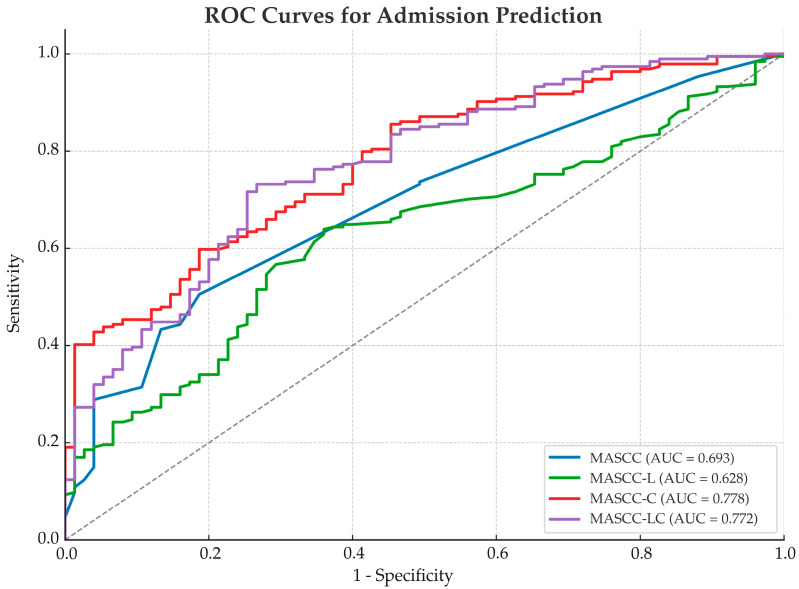
ROC curves comparing the performance of the MASCC and modified models for hospital admission prediction. AUC: area under the curve; MASCC-L = lactate/MASCC; MASCC-C = CRP/MASCC; MASCC-LC = (CRP × lactate)/MASCC. Sensitivity represents the true positive rate; 1–specificity represents the false positive rate.

For the prediction of hospital admission (ward or ICU admission versus discharge), the MASCC-C and MASCC-LC models demonstrated higher AUC values (0.778 and 0.772, respectively) compared to the original MASCC (0.693) and MASCC-L (0.628) models. The MASCC-LC model showed the highest sensitivity (73%) and moderate specificity (73%), while the MASCC-C model demonstrated the highest specificity (81%) among the models analyzed (Table 3, Figure 1).

The precision of these AUC estimates was supported by narrow bootstrap-derived confidence intervals.

**Figure 2:**
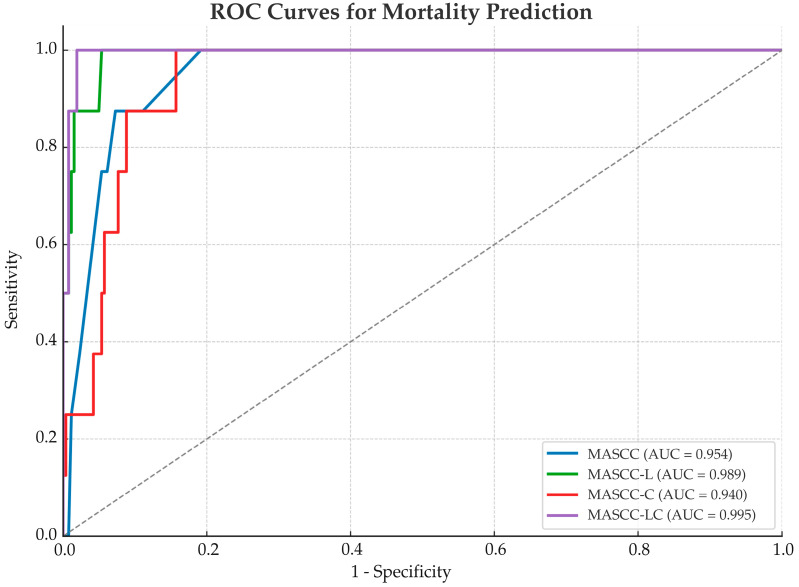
ROC curves comparing the performance of the MASCC and modified models for 30-day mortality prediction. AUC: area under the curve; MASCC-L = lactate/MASCC; MASCC-C = CRP/MASCC; MASCC-LC = (CRP × lactate)/MASCC. Sensitivity represents the true positive rate; 1–specificity represents the false positive rate.

In the ROC analysis of the 30-day mortality predictions, the MASCC-LC model demonstrated the highest discriminative performance with an AUC of 0.995 (95% CI: 0.985–1.000), followed by the MASCC-L (AUC: 0.989; 95% CI: 0.970–0.999), MASCC (AUC: 0.954; 95% CI: 0.910–0.985) and MASCC-C models (AUC: 0.940; 95% CI: 0.891–0.977). All the models achieved 100% sensitivity, with the MASCC-LC model reaching the highest specificity (98%) and positive predictive value (62%) (Table 4, Figure 2).

Despite the limited number of mortality events, the tight bootstrap confidence intervals suggest that the discriminatory power of the models remained statistically robust.

## 4. Discussion

Febrile neutropenia is a clinical syndrome frequently observed in immunocompromised oncology patients and carries a substantial risk of severe complications and mortality. Timely and accurate risk stratification is therefore essential for guiding initial management decisions, optimizing resource allocation and improving patient outcomes, as highlighted in recent validation studies evaluating alternative and modified scoring systems [18,19]. Although the MASCC score is a valuable tool for identifying low-risk patients, its reliance on subjective criteria and exclusion of laboratory parameters limit its sensitivity, especially in the non-low-risk group [7,10]. These limitations may lead to misclassification, suboptimal triage decisions and delays in critical interventions [10,12].

The limitations of the MASCC score become more evident in patients with scores below 21. In the large dataset reported by Klastersky et al., the complication rate was 79% and the mortality rate was 36% in patients with MASCC scores below 15 [20]. These findings indicate that patients with low scores have a high risk of developing sepsis and organ failure and that risk stratification based solely on clinical findings is insufficient. In a meta-analysis by Zheng et al., the sensitivity and specificity of the MASCC score at the threshold of <21 were reported to be 55.6% and 86.0%, respectively, further supporting the inadequacy of using the score alone in clinical decision-making [7].

To address this gap, we developed three new models by integrating lactate and CRP biomarkers into the MASCC score and analyzed their performance in predicting both hospital admission and the 30-day mortality. The MASCC-LC model achieved the highest discriminative performance for mortality with an AUC of 99.5%. While all the models demonstrated 100% sensitivity, the MASCC-LC model stood out with 98% specificity and a positive predictive value of 62%. For the prediction of hospital admission, the MASCC-C model provided 81% specificity and yielded AUC values comparable to those of the MASCC-LC model. These results suggest that incorporating the consideration of simple, objective biomarkers enhances the predictive utility of clinical scores in both directions, identifying not only low-risk patients suitable for outpatient care but also high-risk cases requiring escalation. Previous efforts [21,22] to enhance MASCC-based risk stratification with additional biomarkers such as the presepsin, procalcitonin or angiopoietin ratios have yielded promising but inconsistent results, often limited by small cohorts, heterogeneous methodologies or a lack of external validation.

In this context, biomarkers such as CRP and lactate, which indicate systemic inflammation and tissue hypoperfusion, may offer valuable contributions to risk stratification. For example, in a study conducted by Zhang et al. in patients with small-cell lung cancer (*n* = 216), the integration of the IL-6 and CRP levels with the MASCC score was found to independently predict the development of febrile neutropenia and significantly improved risk estimation [23]. Similarly, in a 2025 prospective observational study by Dimitrijević et al., a CRP/albumin ratio > 2.74 was identified as an independent predictor of the 28-day mortality and demonstrated 33.3% higher predictive power compared to the MASCC score [24]. Additionally, in a study by Klastersky et al., the lactate levels successfully predicted the risk of septic shock, while CRP was significantly associated with mortality [20].

These findings support the results of our study. In a similar manner, a study by Combariza et al. in patients with hematologic malignancies reported that the 30-day mortality reached up to 36% in patients with MASCC scores < 21 and CRP levels > 15 mg/dL, while the rate was 0% among low-risk patients with CRP < 15 mg/dL [11]. Sütcüoğlu et al. integrated serum uric acid into the MASCC score and showed that the 30-day mortality reached 50% when both the parameters were elevated [8]. This suggests that biomarker-based modifications are not limited to CRP and lactate and can be supported by indicators from different biological systems. Furthermore, in a 2025 prospective study conducted by Jakobac et al. in patients with febrile neutropenia and hematologic malignancies, the presepsin (PSP) levels were found to have higher sensitivity than CRP and procalcitonin in distinguishing septic complications. Each 1 ng/mL increase in PSP was associated with a 5% increase in the mortality risk, and elevated PSP levels were significantly associated with the one-year mortality [25].

Procalcitonin (PCT) is another promising biomarker for predicting sepsis in febrile neutropenia. Chaftari et al. showed that PCT > 0.25 ng/mL combined with lactate > 2.2 mmol/L significantly increased the 14-day mortality and intensive care unit admission and was more successful than the MASCC score in predicting bacteremia [10]. Similarly, Yadav et al. demonstrated that elevated serum PCT levels at admission are an independent predictor of the 30-day mortality among patients with febrile neutropenia, further supporting the prognostic utility of using infection-related biomarkers beyond clinical scores [13]. In a separate approach, a study by Choi et al. emphasized that nomograms developed using CBC-based parameters (e.g., the mean platelet volume—MPV) and qSOFA scores produced results comparable to those of the MASCC score [26].

In addition, the modified MASCC model (PACI model) proposed by Viana et al. excluded complex infections and increased the specificity from 85% to 100% [27]. This study demonstrates that the effectiveness of risk scoring systems can be improved not only by integrating new parameters but also by refining existing structures to better align with clinical objectives.

In this study, the clinical stability reflected by the MASCC score and the representation of systemic inflammation and tissue perfusion disturbances by CRP and lactate were mathematically integrated using ratio- and multiplication-based approaches to create new models. In this respect, our study offers a novel and practical method that differs from the existing modifications in the literature.

The MASCC-LC model offers practical advantages beyond its prognostic performance. With only three parameters (CRP, lactate and MASCC score), it is computationally simple and based on data available within 30–60 min of emergency department presentation. For example, point-of-care lactate testing reduced the turnaround times from around 53 to 33 min in one study [28]. This facilitates its integration into rapid triage protocols, particularly in overcrowded or resource-limited settings. Moreover, its simplicity makes it an ideal candidate for embedding into electronic clinical decision support systems, enabling automated alerts for high-risk patients and streamlining early intervention, as demonstrated by the widespread use of sepsis CCDS tools embedded in electronic health records [29].

While the original MASCC score was designed to identify patients suitable for outpatient management, the MASCC-LC model broadens its clinical utility by accurately predicting both hospitalization and mortality. This aligns with current clinical priorities, as emphasized by Alsharawneh et al., which advocate for early identification of clinical deterioration to guide timely and proactive interventions in emergency settings [30]. Rather than serving as a replacement, the model complements existing tools by enhancing their discriminatory power. Its high sensitivity and near-perfect AUC values suggest that it can act as a dual-purpose instrument that supports both safe discharge decisions and early escalation of care when necessary. Although we did not assess the clinical implementation outcomes in this study, future research may explore real-time integration of the MASCC-LC model into emergency department workflows and evaluate its impact on triage decisions and patient outcomes.

In conclusion, our study demonstrates that biomarker-based modified MASCC models are significantly superior to the original MASCC score in predicting both mortality and hospital admission. In particular, the MASCC-LC model, with its use of only three parameters and 100% sensitivity, has the potential to be easily integrated into clinical decision-making in resource- and time-limited settings such as the emergency department. However, external validation of these models through larger, multicenter and prospective studies is necessary. This will allow for the development of more objective, reliable and patient-centered risk stratification systems in the management of febrile neutropenia.

### Study Limitations

This study has several limitations. Its retrospective design and single-center setting inherently limit the broader applicability and generalizability of the findings to other institutions and patient populations. Additionally, although the sample size was adequate, the relatively low number of mortality events (*n* = 8; 3%) may have limited the statistical reliability of the mortality-related performance measures, despite the high AUC values. Another limitation is the absence of a calibration analysis, which would have complemented the ROC-based discrimination evaluation by assessing how well the predicted probabilities align with the actual outcomes. Since the frequency and methods of measuring the CRP and lactate levels may vary across institutions, the use of these biomarkers may not be equally feasible in all clinical environments. Finally, the MASCC-LC model developed in this study has not yet been tested in independent external validation studies and therefore its applicability requires confirmation in multicenter prospective settings before it is adopted in clinical practice. In addition, variability in the availability or turnaround time of CRP and lactate testing across emergency departments may limit the universal applicability of the MASCC-LC model.

## 5. Conclusions

This study demonstrates that new models generated by mathematically integrating the MASCC score with biomarkers such as lactate and CRP can predict the 30-day mortality and hospital admission requirements with greater accuracy than the classical MASCC score in patients with febrile neutropenia. In particular, the MASCC-LC model, which can be easily calculated using only three parameters and provides high sensitivity, stands out as an effective decision support tool in time- and resource-constrained clinical environments such as emergency departments. The findings suggest that biomarker-based modified scores may contribute to more objective, reliable and patient-centered risk stratification approaches in the management of febrile neutropenia.

## Figures and Tables

**Table 1:** Demographic and clinical characteristics and outcomes of study population.

		Value (Mean ± SD)
**Age**		67.6 ± 12.4
**MASCC Score**		20.4 ± 4.4
**CRP (mg/L)**		138.5 ± 107.8
**Lactate (mmol/L)**		2.6 ± 2.5
		***n* (%)**
**Outcome**	Discharged	75 (27.9)
	Ward Admission	150 (55.8)
	ICU Admission	44 (16.4)
**Mortality**	None	261 (97.0)
	Present	8 (3.0)

MASCC: Multinational Association of Supportive Care in Cancer; CRP: C-reactive protein; ICU: intensive care unit; SD: standard deviation.

**Table 2:** Comparison of clinical parameters across outcome and mortality groups in febrile neutropenia.

Parameter	Outcome (Mean ± SD)				Mortality (Mean ± SD)		
	Discharged	Ward Admission	ICU Admission	*p* †	None	Present	*p* ‡
**Age**	66.9 ± 12.9	67.7 ± 12.6	68.6 ± 11.2	0.737	67.6 ± 12.5	68.9 ± 7.4	0.991
**MASCC Score**	22.5 ± 2.9	20.9 ± 3.6	15.0 ± 4.7	**<0.001**	20.7 ± 4.1	11.0 ± 2.6	**<0.001**
**CRP**	73.6 ± 66.8	149.7 ± 106.3	211.3 ± 111.2	**<0.001**	135.4 ± 107.7	241.8 ± 38.4	**0.001**
**Lactate**	2.1 ± 1.0	2.1 ± 1.0	5.4 ± 5.0	**<0.001**	2.4 ± 1.5	11.5 ± 8.4	**<0.001**

MASCC: Multinational Association of Supportive Care in Cancer; CRP: C-reactive protein; ICU: intensive care unit; SD: standard deviation. † Mann–Whitney U test. ‡ Kruskal–Wallis test.

**Table 3:** Predictive performance of scoring models for hospital admission.

Scoring Model	AUC	SE	95% CI		Sensitivity	Specificity	PPV	NPV
			Lower Limit	Upper Limit				
**MASCC**	0.693	0.034	0.623	0.763	0.51	0.81	0.88	0.39
**MASCC-L**	0.628	0.036	0.559	0.697	0.64	0.64	0.82	0.41
**MASCC-C**	0.778	0.028	0.721	0.831	0.6	0.81	0.89	0.44
**MASCC-LC**	0.772	0.029	0.712	0.830	0.73	0.73	0.88	0.51

AUC: area under curve; SE: standard error; CI: confidence interval; PPV: positive predictive value; NPV: negative predictive value; MASCC: Multinational Association of Supportive Care in Cancer, MASCC-L: lactate-integrated MASCC score; MASCC-C: CRP-integrated MASCC score; MASCC-LC: combined lactate- and CRP-integrated MASCC score.

**Table 4:** Predictive performance of scoring models for mortality.

Scoring Model	AUC	SE	95% CI		Sensitivity	Specificity	PPV	NPV
			Lower Limit	Upper Limit				
**MASCC**	0.954	0.019	0.910	0.985	1.00	0.81	0.14	1.0
**MASCC-L**	0.989	0.008	0.970	0.999	1.00	0.95	0.36	1.0
**MASCC-C**	0.940	0.022	0.891	0.977	1.00	0.84	0.16	1.0
**MASCC-LC**	0.995	0.004	0.985	1.000	1.00	0.98	0.62	1.0

AUC: area under curve; SE: standard error; CI: confidence interval; PPV: positive predictive value; NPV: negative predictive value; MASCC: Multinational Association of Supportive Care in Cancer, MASCC-L: lactate-integrated MASCC score; MASCC-C: CRP-integrated MASCC score; MASCC-LC: combined lactate- and CRP-integrated MASCC score.

## Data Availability

The raw data supporting the conclusions of this article will be made available by the authors on request (Efe Kanter, efekanter@hotmail.com).

## Abbreviations

The following key abbreviations are used throughout this manuscript:

MASCC	Multinational Association for Supportive Care in Cancer
MASCC-L	Lactate-integrated MASCC score
MASCC-C	CRP-integrated MASCC score
MASCC-LC	Combined lactate- and CRP-integrated MASCC score
